# Validity of sports watches when estimating energy expenditure during running

**DOI:** 10.1186/s13102-017-0089-6

**Published:** 2017-12-20

**Authors:** Lilian Roos, Wolfgang Taube, Nadja Beeler, Thomas Wyss

**Affiliations:** 1 Section for Elite Sport, Swiss Federal Institute of Sport Magglingen SFISM, Hauptstrasse 247, 2532 Magglingen, Switzerland; 2 Department of Medicine, Movement and Sport Science, University of Fribourg, Boulevard de Pérolles 90, 1700 Fribourg, Switzerland

**Keywords:** Wearables, High-intensity, Maximal accumulated oxygen deficit, Validation, Monitoring training

## Abstract

**Background:**

The aim of this study was to assess the accuracy of three different sport watches in estimating energy expenditure during aerobic and anaerobic running.

**Methods:**

Twenty trained subjects ran at different intensities while wearing three commercial sport watches (Suunto Ambit2, Garmin Forerunner920XT, and Polar V800). Indirect calorimetry was used as the criterion measure for assessing energy expenditure. Different formulas were applied to compute energy expenditure from the gas exchange values for aerobic and anaerobic running.

**Results:**

The accuracy of the energy expenditure estimations was intensity-dependent for all tested watches. During aerobic running (4–11 km/h), mean absolute percentage error values of −25.16% to +38.09% were observed, with the Polar V800 performing most accurately (stage 1: −12.20%, stage 2: −3.61%, and stage 3: −4.29%). The Garmin Forerunner920XT significantly underestimated energy expenditure during the slowest stage (stage 1: −25.16%), whereas, the Suunto Ambit2 significantly overestimated energy expenditure during the two slowest stages (stage 1: 38.09%, stage 2: 36.29%). During anaerobic running (14–17 km/h), all three watches significantly underestimated energy expenditure by −21.62% to −49.30%. Therefore, the error in estimating energy expenditure systematically increased as the anaerobic running speed increased.

**Conclusions:**

To estimate energy expenditure during aerobic running, the Polar V800 is recommended. By contrast, the other two watches either significantly overestimated or underestimated energy expenditure during most running intensities. The energy expenditure estimations generated during anaerobic exercises revealed large measurement errors in all tested sport watches. Therefore, the algorithms for estimating energy expenditure during intense activities must be improved before they can be used to monitor energy expenditure during high-intensity physical activities.

## Background

The amount of energy spent on a specific activity – commonly known as energy expenditure (EE) – is important not only for athletes but also for patients suffering from obesity or diabetes [1–3]. The term EE is often used with regard to nutrition, sport science, occupational tasks, and athlete training, areas in which it is important to monitor the demands of various physical activities. Especially in clinical nutrition settings (e.g. monitoring the exercise activity of obese people), it is important to use devices that provide accurate EE measurements as these measurements are crucial in determining the amount of calories that a patient can consume without gaining weight [3]. Similarly, active and lean people may be interested in obtaining precise EE data during their training sessions. Therefore, devices that can accurately measure EE are useful.

Indirect calorimetry can be performed by using stationary or portable spirometers to measure breath-by-breath gas exchange, which in turn is analyzed in order to estimate EE. This reference method measures activities performed over a duration of 1–3 h and has been found to be accurate during rest periods and various levels of exercise intensity [4, 5]. Indirect calorimetry is considered the most feasible method for attaining accurate data for short-term physical activity in a laboratory setting [6]. Another option is to estimate EE using heart rate (HR) data, due to the linear relationship of oxygen consumption and HR [7]. Previous findings supported HR measurements to be a valid method to assess EE in a laboratory or field setting, EE estimations were even better when using percentage of HR reserve or difference between active and resting HR [8]. When considering different methods for assessing EE, it becomes obvious that there is a trade-off between accuracy, feasibility, and costs [9]. At the same time, factors such as device usability and movement constraints are important to consider. For example, sports watches could constitute the perfect solution as they are user-friendly, relatively low-priced, non-invasive, and can provide other important information during a training session, such as duration, HR, speed, distance and altitude covered [10, 11]. It is important to understand how accurate sports watches are in assessing EE during varying levels of exercise intensity. For researchers to make informed decisions about which products to include in a study or trial. This information is equally relevant for professional and recreational athletes who use the popular sports watches to monitor different variables during their training sessions. However, the accuracy of the newest sports watches (season 2015) in assessing EE is thus far unknown. The companies developing these devices use proprietary algorithms to estimate EE. Generally, these algorithms consider variables such as age, weight, height, sex, maximal heart rate (HR_max_), and maximal oxygen uptake (VO_2peak_) in computing an individual’s EE. A recent study reported that prediction accuracy of EE during running was significantly increased when real-time running speed was included [12]. The newer generation of sports watches also have built-in accelerometers, so it is likely that acceleration data is factored into the algorithm as well. Even some earlier devices from different manufacturers had accelerometers implemented. However, sports watch developers prefer to keep their algorithms secret, and there exists only limited published research regarding the development, validity, and reliability of EE estimation algorithms in sports watches [8, 10, 13], especially with regard to vigorous physical activity and the inclusion of accelerometer data into the algorithms. Therefore, this study aims to validate the EE estimations of three sports watches (Suunto Ambit2, Garmin Forerunner920XT, and Polar V800), as these manufacturers are the top competitors on the market, during low, moderate, and high-intensity running against estimates of EE from indirect calorimetry as the criterion measure.

## Methods

### Study design

Each participant visited the lab twice. The visits were at least 2 days but no more than 2 weeks apart and took place at the same hour of the day. The participants were asked to avoid intense and strenuous training the day before the tests. Furthermore, the participants were asked to abstain from alcohol 24 h and from food and drinks with caffeine for the 6 h before each test.

During their first visit, the athletes were informed about the study procedures, anthropometric data were measured, and the preliminary test was performed. The height and weight measurements were taken to the nearest 0.01 m using a stadiometer and to the nearest 0.01 kg using a calibrated scale (Model 213 and Model 877, respectively; seca GmbH, Hamburg, Germany). The two running trials were performed on a treadmill (Model Mercury, h/p/cosmos sports & medical GmbH, Nussdorf-Traunstein, Germany) with an increment of 1% to simulate outdoor running [14]. First, the participants participated in a submaximal incremental exercise test of maximally ten 5 min stages, starting at 5 km/h and with an incremental increase of 1.5 km/h per stage [15, 16]. The test was stopped when the participants reached a respiratory exchange ratio (RER) of ≥1.0 (mean over 1 min). Afterwards, the participants rested for 8 min. Second, the participants performed an all-out test to assess their HR_max_ and VO_2peak_. The all-out test started at 7 km/h, the first three stages lasted 1 min each, and the incremental increase was 1 km/h. The following stages lasted 30 s each, with 0.5 km/h incremental increases until volitional exhaustion [17]. During the last 15 s of each running stage, the participants were asked to rate their perceived exertion on a Borg scale ranging from 6 to 20 [18]. From the speed at VO_2peak_ (vVO_2peak_), the individual’s relative speeds for the test on the second visit were calculated at 30%, 50%, 70%, 90%, and 110% of vVO_2peak_. To measure breath-by-breath automatic gas exchange, the Moxus Modular Metabolic System (AEI Technologies, Pittsburg PA, USA) was used. Several authors previously validated the Moxus Modular Metabolic System against the Douglas bag method and reported adequate to high reliability and reasonable validity during submaximal and maximal activities [4, 19].

On the second testing day, the participants were each fitted with three sports watches (Suunto Ambit2, Suunto Oy, Vantaa, Finland; Garmin Forerunner920XT, Garmin International Inc., Olathe KS, USA; Polar V800, Polar Electro Oy, Kempele, Finland) – and their corresponding HR monitors. The watches were set according to each individual’s age, height, weight, HR_max_, and sex (Polar V800 only). The participants wore all three watches at the same time. Each participant wore two watches on the left wrist and forearm, the third watch on the right wrist, and the heart rate monitors (paired with the corresponding watch) around the chest. The positioning of the watches and the localization of the paired heart rate monitors was randomized. First, the participants were asked to stand still on the treadmill for 2 min, during which a baseline measurement was taken before the treadmill test began. The first three stages were performed at individual running speeds of 30%, 50%, and 70% of vVO_2peak_ and lasted 10 min each, with a 2 min standing break in-between the stages. The last two stages, performed at 90% and 110% of vVO_2peak,_ lasted 90 s each, with the same standing break in-between. All measurement devices were calibrated before each test and used in accordance with the manufacturer recommendations. The training profile “running” and for Garmin Forerunner920XT “indoor running” was selected from each watch’s menu. The watches were simultaneously started and stopped directly before and after each stage. The data were saved on the watch and synchronized using the proprietary online software (Suunto Movescount, Suunto Oy, Vantaa, Finland; Garmin Connect, Garmin International Inc., Olathe KS, USA; Polar Flow, Polar Electro Oy, Kempele, Finland) on a computer after each test. From there, the individual caloric values from the five stages were transferred to a database for further analysis.

### Participants

Twenty healthy participants (12 males and 8 females) volunteered to participate in this study (age 23.90 ± 1.92 years, height 1.74 ± 0.08 m, weight 66.90 ± 10.02 kg, HR_max_ 193.10 ± 4.88 bpm, VO_2peak_ 55.75 ± 7.33 ml/min/kg). All participants were recreational or competitive runners, and none of them had experienced any injury to their lower extremities within the past year. Before the first test, the participants were informed about the procedure and aims of the study and signed a written informed consent form that had been previously approved by the Institutional Review Board of the Swiss Federal Institute of Sport Magglingen. This study meets the principals outlined in the Declaration of Helsinki.

### Data analysis – EE estimation during low to moderate running intensity

All the data from the watches was normalized to the unit of kcal/min. Missing values resulting from unsystematic HR monitor failure or malfunction were replaced using the relative difference (slope) from the reference mean to the specific watch mean from the corresponding running stage. For the EE measurements from the criterion measure, the formula of Elia and Livesey [20] was used to compute the total EE from the gas exchange data in kcal/min for the three submaximal categories (stage 1: 30% vVO_2peak_, stage 2: 50% vVO_2peak_, and stage 3: 70% vVO_2peak_). These formulas are commonly accepted for estimating EE during aerobic or submaximal intensities [6, 20–25]. However, very few studies have validated these formulas for anaerobic activities.

### Data analysis – EE estimation during high-intensity running

The few studies that have examined high-intensity exercises generally reported low validity with regard to the criterion measure of indirect calorimetry [6, 26, 27]. Therefore, other methods were needed to overcome these measurement problems during vigorous physical activity. Medbo and colleagues [15] first proposed a new way to assess anaerobic proportions of EE during high-intensity physical activities. By assuming a linear relationship between running speed and oxygen uptake, they were able to interpolate to intensities greater than the maximal oxygen uptake [15]. From the intrapolated value at a certain speed or intensity, the measured oxygen consumption can be subtracted. The difference, integrated over the duration of the activity, can be used to estimate the maximal accumulated oxygen deficit (MAOD). Several authors reported MAOD to be the most accurate, non-invasive method for determining the anaerobic proportion of EE during high-intensity activities [16, 28, 29]. Therefore, the MAOD method was applied to compute the difference between the measured breath-by-breath gas exchange and the theoretically necessary oxygen uptake [15, 28] for the near-maximal and the supramaximal categories (stage 4: 90% vVO_2peak_ and stage 5: 110% vVO_2peak_). Considering the high intensity of these two bouts and the measured RER values of ≥1.0 following these exercises, pure carbohydrates can be assumed as the muscle energy source. Therefore, the oxygen values, measured in ml/min, were multiplied by 5.04 kcal/l oxygen [25, 30].

### Statistical analysis

The data were tested for normality using the Shapiro-Wilk test and mean values and standard deviations (SD) were calculated. The data were analyzed using a repeated-measures ANOVA with a Bonferroni post-hoc analysis. The validity of the three watches was initially investigated using Pearson’s correlation analyses. Furthermore, mean absolute error (MAE) and mean absolute percentage error (MAPE) of each watch compared to the criterion measure were calculated. As the threshold for accurate EE estimations, a MAPE ≤10% was defined, similar to the definition used by other researchers [11, 31]. The individual error, which was used specifically to assess inter-individual differences, was computed with the root mean square error (RMSE). Bland-Altman plots including 95% limits of agreement (±1.96 times SD) with their corresponding intercept and slope were created to graphically represent the data and to visualize systematic differences in EE estimation [32]. The level of significance was set at *p* < 0.05, and the statistical analyses were performed using SPSS 23 (IBM Corporation, Armonk NY, USA).

## Results

Nineteen participants completed both the first and second test. One male athlete could not finish the last two stages due to a cold and, therefore, all his data were excluded from the analysis. Due to technical issues, 10 EE files (3.51%) from the watches had to be replaced using relative estimated data. The descriptive data from the criterion measure and the three sports watches are presented in Table 1. The measured EE generally increased from stage to stage.

**Table 1:** Total energy expenditure measured per device including HR and speed per stage

	30% vVO_2peak_	50% vVO_2peak_	70% vVO_2peak_	90% vVO_2peak_	110% vVO_2peak_
Criterion measure [kcal/min]	3.89 ± 0.94	7.84 ± 2.05	11.72 ± 2.70	16.95 ± 4.09	21.07 ± 4.76
Suunto Ambit2 [kcal/min]	5.17 ± 1.25*	10.60 ± 3.14*	13.65 ± 2.18	12.97 ± 2.25*	14.02 ± 2.08*
Garmin Forerunner 920XT [kcal/min]	2.89 ± 0.81*	7.39 ± 2.48	10.63 ± 2.42	10.38 ± 2.27**	10.67 ± 2.84**
Polar V800 [kcal/min]	4.32 ± 1.20	7.45 ± 1.81	11.15 ± 2.50	11.75 ± 2.70*	12.70 ± 3.08**
HR [bpm]	99.84 ± 17.95	129.24 ± 20.30	150.80 ± 12.54	167.34 ± 7.81	176.35 ± 7.66
Speed [km/h]	4.69 ± 0.69	7.83 ± 1.15	10.93 ± 1.62	14.05 ± 2.07	17.06 ± 2.28

Values are expressed as mean ± standard deviation

*vVO*_*2peak*_ speed at maximal oxygen uptake, *HR* heart rate, *bpm* beats per minute

*significantly different from criterion measure (*p* < 0.05)

**significantly different from criterion measure (*p* < 0.001)

### EE estimation during low and moderate intensity running

**Fig. 1:**
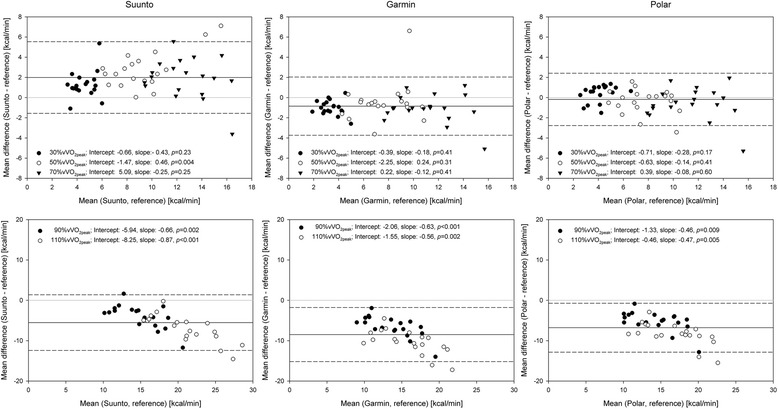
Bland-Altman plots for Suunto Ambit2, Garmin Forerunner920XT, and Polar V800. Presented in groups for low to moderate running intensities (30%, 50%, and 70% vVO2peak) and high-intensity running (90% and 110% vVO2peak). The mean bias is marked as a solid black line, and ±1.96 times standard deviation as dashed black lines. vVO2peak: speed at maximal oxygen uptake

The Pearson’s correlation analysis revealed significantly correlated data between the reference values and the EE values from each watch for the first three stages (*r* = 0.63–0.85, *p* < 0.05), except for the Suunto watch during the first stage (*r* = 0.30, *p* = 0.22). The MAE, MAPE, and RMSE for all running intensities are presented in Table 2. For the Garmin watch, the underestimated value of EE during the first stage was significantly different (*p* = 0.01) from the EE measured by the criterion measure. In contrast, the Suunto Ambit2 significantly overestimated EE during stage 1 (*p* = 0.002) and stage 2 (*p* = 0.003). In Fig. 1, the data of each tested watch and the reference method are presented using Bland-Altman plots. For the low to moderate running intensities overall, the mean bias (±1.96 SD) was 1.99 (−1.56; 5.54) kcal/min for the Suunto Ambit2, −0.85 (−3.73; 2.04) kcal/min for the Garmin Forerunner920XT, and −0.18 (−2.77; 2.41) kcal/min for the Polar V800 (Fig. 1). No systematic errors were observed during the low to moderate intensity running, except for the Suunto Ambit2 during stage 1 (*p* = 0.004; Fig. 1).

**Table 2:** Concurrent validity (tested device vs. criterion measure) of the three sports watches

	30% vVO_2peak_	50% vVO_2peak_	70% vVO_2peak_	90% vVO_2peak_	110% vVO_2peak_
Mean difference [%]					
Suunto Ambit2	38.09 ± 42.06	36.29 ± 20.57	19.16 ± 18.03	−21.62 ± 12.54	−31.85 ± 10.54
Garmin Forerunner920XT	−25.16 ± 16.09	−4.87 ± 29.34	−8.83 ± 10.50	−37.94 ± 8.82	−49.30 ± 9.18
Polar V800	12.20 ± 21.65	−3.61 ± 15.04	−4.29 ± 12.02	−29.98 ± 9.68	−39.52 ± 8.89
MAE [kcal/min]					
Suunto Ambit2	1.46	2.75	2.32	4.15	7.05
Garmin Forerunner920XT	1.05	1.28	1.39	6.56	10.40
Polar V800	0.86	0.89	1.21	5.20	8.37
MAPE [%]					
Suunto Ambit2	41.93	36.30	21.32	23.05	31.85
Garmin Forerunner920XT	26.28	17.59	11.54	37.94	49.30
Polar V800	22.76	11.43	10.09	29.98	39.52
RMSE [kcal/min]					
Suunto Ambit2	1.82	3.27	2.74	4.88	7.81
Garmin Forerunner920XT	1.22	1.98	1.77	7.05	10.81
Polar V800	0.93	1.26	1.67	5.75	8.81

Results are expressed as mean ± standard deviation

*vVO*_*2peak*_ speed at maximal oxygen uptake, *MAE* mean absolute error, *MAPE* mean absolute percentage error, *RMSE* root mean square error

### EE estimation during high-intensity running

The Pearson’s correlation analysis revealed significantly correlated data between the reference values and the values from each watch at every stage (*r* = 0.72–0.82, *p* < 0.001). During the last two stages (90% vVO_2peak_ and 110% vVO_2peak_), the EE values from all the watches were significantly lower compared to the EE values measured by the criterion measure (all *p* < 0.001; Table 2) and this underestimation grew as the running intensity increased. In Fig. 1, a proportional error leading to a greater EE underestimation as the running speed increased is reported. The Suunto watch displayed a mean bias (±1.96 SD) of −5.51 (−12.41; 1.38) kcal/min, the Garmin device of −8.48 (−15.18; −1.78) kcal/min, and the Polar device of −6.79 (−12.85; −0.73) kcal/min.

## Discussion

### EE estimation during low and moderate intensity running

The results of this study demonstrated near acceptable validity based on the MAPE for two of the three sports watches, the Polar V800 and the Garmin Forerunner920XT, during the moderate running stages, with the Polar V800 presenting as the most valid and accurate watch of the three compared devices (Fig. 1). The Garmin and Polar sports watches performed best during stages 2 and 3, during which the smallest MAPE values were observed (17.59% and 11.54% vs. 11.43% and 10.09%, respectively). The EE values of the Polar watch did not differ statistically from those of the criterion measure during the slowest two stages, 30% and 50% vVO_2peak_. However, the MAPE of 22.76% and 11.43% were greater than the previously defined range of 10% deviance from the criterion measure [11, 31]. The Garmin Forerunner920XT significantly underestimated the EE during the slowest stage (*p* = 0.01). On the contrary, the Suunto Ambit2 significantly overestimated EE during stages 1 and 2 (*p* = 0.002 and *p* = 0.003, respectively). During stage 3, no statistical difference was observed between the values provided by the Suunto watch and the criterion measure, however the MAPE of 21.32% was greater than the acceptable error margin. Despite the fact that all the watches used similar individual information regarding weight, height, and HR_max_, each device used a different algorithm, thus explaining the slight differences between the watches. A recent study reported increased accuracy in EE estimation when running speed was included in the equation [12]. As the running in the present study was performed on an indoor treadmill, the signal of the global positioning system (GPS) was not fully reliable. Further, due to the previously mentioned EE estimation algorithm non-disclosure of each company, it is unclear if and how measured (GPS or accelerometer based) running speed was included in the watches EE algorithm. Spierer and colleagues [13] validated an accelerometer and HR-based device (Actiheart, CamNtech Ltd., Cambridge, United Kingdom) and reported a MAPE of 41%, 17%, and 24% for EE estimations based on accelerometer, HR, and combined accelerometer/HR data, respectively. The smallest MAPE was observed for the algorithm using HR only. Despite the similar approach used for estimating EE, only running speeds from 4.0 and 7.2 km/h were investigated in the previous study [13]. These speeds are comparable to stages 1 and 2 in the current study. Especially during stage 2, smaller MAPE values were observed for the Polar V800 and Garmin Forerunner920XT. Several other studies investigated EE estimation in different accelerometer based devices compared to indirect calorimetry as a criterion in recent years. The smallest differences were generally observed during moderate waking and running exercises [33–35]. Generally, the mean differences during rest, slow walking, and intermittent sport activities were, however, greater compared to studies with HR based EE estimations [13, 33, 34, 36]. Hongu et al. [10] examined wrist worn sports watches from Garmin and Polar reported significant differences in EE estimations and poor reliability compared to the values provided by the criterion measure at speeds of 7 km/h. However, only speeds from slow to brisk walking were investigated, and the criterion measure was accelerometer-based; therefore, the comparison to the current study is limited, despite the fact that similar types of sports watches were investigated. A recent study that investigated EE measures of seven different wrist-worn devices during walking and running speeds from 4.0 to 11.1 km/h reported MAPE between 25 and 35% [37]. These results were greater than the values for the Polar V800 and Garmin Forerunner920XT in the present study, as the EE values given by Garmin and Polar watches did not significantly differ (*p* > 0.05) from those given by the criterion measure at lower speeds from 7 to 11 km/h. A reason for the improved results in the current study is likely a consequence of the ongoing efforts of the manufacturers and developers to improve the applied EE algorithms.

### EE estimation during high-intensity running

The greatest MAPE values (range 29.98–49.31%) were reported for the two most intense running stages 4 and 5, with the exception of the Suunto Ambit2 (see Table 2). For the Suunto watch, the MAPE values for the five stages ranged between 21.32–41.93%, with the smallest value observed for the stage 3. Hence, the accuracy of EE estimation by the Suunto Ambit2 must be regarded with caution, as it overestimated EE during low to moderate intensities and underestimated EE at higher intensities. The RMSE results were similar to the MAE values for all watches and running intensities (Table 2). Therefore, it can be concluded that the errors did not differ greatly between the participants, and the devices seem to perform consistently when used by different runners [31]. The Bland-Altman plots of the two high-intensity running stages showed a similar proportional error in all three sports watches. The more intense the activity, the greater the underestimation of EE in the Suunto, Garmin, and Polar watches (Fig. 1). This confirms the findings of previously published validation studies that included running stages at high speeds up to 17 km/h and reported that a proportional error was observed with increasing exercise intensity [26, 27, 38]. Koehler et al. [26] performed a treadmill running validation study with trained participants and observed a MAPE of up to 36% in EE estimation with an acceleration-based device worn on the upper arm. Although the reported MAPE is comparable to those of the current study, the devices used different approaches to estimate EE: the devices by Koehler et al. [26] used acceleration and heat flux, while the devices in the present study are HR-based, with an unknown contribution of accelerometer data. The same device was validated in another study on daily life activities; an overall MAPE value of 9% was observed [11]. However, Lee et al. [11] presented no data for solely walking or running, and therefore the comparison with the present study is limited. Generally, previously published studies using wearable devices reported acceptable to good validity with regard to EE values at rest and during activities of low to moderate intensity or at speeds below 10 km/h. However, accuracy decreased and MAPE increased as the intensity of the activities performed increased [13, 26, 27, 38].

The current study’s results showed that the EE estimation formulas used in the Suunto Ambit2, Garmin Forerunner920XT, and Polar V800 sports watches are inaccurate at high intensities that correspond to combined aerobic and anaerobic metabolism with larger parts of the aerobic metabolism. Therefore, sports watch developers must improve the existing algorithms to counteract this estimation error. Especially, because certain users of such devices, e.g. elite athletes and coaches, are very interested in accurate and valid data from the most often used devices during their daily training sessions. A possibility might be trying to detect or estimate the anaerobic threshold and using a different algorithm to calculate EE from thereon. However, it is not possible to recommend general thresholds from the current study setting. Furthermore, the accelerometer implemented in the newest generation of sports watches might be of added value when used to correctly detect high-intensity exercise bouts. However, previous research reported slightly decreased EE estimation accuracy when implementing additional measures such as accelerometer data to the existing HR-based algorithm [13]. From a nutritional viewpoint, an underestimation of EE in these devices is preferable for participants trying to lose weight, as food intake should not surmount the amount of energy spent on activities and rest. However, only few validation studies examining high-intensity activities or running at high speeds have been published. Yet, endurance athletes’ training sessions usually consist of low, moderate, and intense bouts and therefore an accurate estimation of EE is necessary across the full intensity range. Hence, the use of these sports watches is currently not recommended for athletes trying to monitor training intensity through EE.

### Strengths and limitations

A positive point of the current study is that the volunteer participants were all trained athletes who were able to perform the running stages at or above their VO_2peak_. Moreover, the running speeds chosen for the five stages ranged from low to supramaximal intensities, hence the three sports watches were tested across a broad range. This is especially important for endurance athletes wishing to use EE to categorize their training intensity. A limitation of the current study is that the MAOD method assumes a linear relationship between running speed and oxygen uptake; this assumption has been challenged before. By assuming a linear relationship at higher intensities, the MAOD might be underestimated as the relationship might become curvilinear [39, 40]. Furthermore, comparisons between studies that use different procedures to estimate the oxygen consumption and intensity relationship are limited. Determination of MAOD is influenced by the testing procedure and was shown to have a relatively poor reproducibility [41, 42]. These disadvantages of MAOD have to be acknowledged when interpreting the current results. However, due to the lack of valid and reliable alternatives, it is currently considered the most feasible method to non-invasively assess anaerobic contributions during intense performance [28]. As every subject performed each stage only once, no reliability measurements could be calculated. However, such data would add value when describing the accuracy of these devices. Finally, only running was investigated, and therefore the results of the current study cannot be generalized for other endurance training methods.

## Conclusions

To conclude, the findings of the present study indicate that the accuracy of the EE estimations provided by the commercial sports watches currently available from Suunto, Garmin, and Polar is intensity-dependent. According to Lee et al. [11] and Nelson et al. [31], MAPE of ≤10% are acceptable for an accurate measurement. Only the Polar V800 met this restriction during the moderate running stage 3 and came close to it during stage 2 with MAPE of 10–11% compared to the criterion measure. Followed by the Garmin Forerunner920XT during stage 3 with a MAPE of 12%. In contrast to the Garmin and Suunto watches, the Polar device did not significantly differ from the criterion measure during any of the first three running stages. However, all three sports watches significantly underestimated EE during the high intensities, with a proportional error increasing as the running speed increased. Hence, the formulas for EE estimation have to be improved to correctly assess the increased EE demands during intense activities.

## Abbreviations

EE: Energy expenditure
GPS: Global positioning system
HR: Heart rate
HR_max_: Maximal heart rate
MAE: Mean absolute error
MAOD: Maximal accumulated oxygen deficit
MAPE: Mean absolute percentage error
RER: Respiratory exchange ratio
RMSE: Root mean square error
SD: Standard deviation
VO_2peak_: Maximal oxygen uptake
vVO_2peak_: Speed at maximal oxygen uptake
